# FGAP: an automated gap closing tool

**DOI:** 10.1186/1756-0500-7-371

**Published:** 2014-06-18

**Authors:** Vitor C Piro, Helisson Faoro, Vinicius A Weiss, Maria BR Steffens, Fabio O Pedrosa, Emanuel M Souza, Roberto T Raittz

**Affiliations:** 1 Laboratory of Bioinformatics, Professional and Technological Education Sector, Federal University of Paraná, Curitiba, PR, Brazil, Rua Dr. Alcides Vieira Arcoverde 1225, Curitiba, Paraná, Brazil; 2 Department of Biochemistry and Molecular Biology, Federal University of Paraná, Curitiba, PR, Brazil, Av. Cel. Francisco H. dos Santos, Curitiba, Paraná, Brazil

**Keywords:** Genome finishing, Gap filling, Gap closure

## Abstract

**Background:**

The fast reduction of prices of DNA sequencing allowed rapid accumulation of genome data. However, the process of obtaining complete genome sequences is still very time consuming and labor demanding. In addition, data produced from various sequencing technologies or alternative assemblies remain underexplored to improve assembly of incomplete genome sequences.

**Findings:**

We have developed FGAP, a tool for closing gaps of draft genome sequences that takes advantage of different datasets. FGAP uses BLAST to align multiple contigs against a draft genome assembly aiming to find sequences that overlap gaps. The algorithm selects the best sequence to fill and eliminate the gap.

**Conclusions:**

FGAP reduced the number of gaps by 78% in an *E. coli* draft genome assembly using two different sequencing technologies, Illumina and 454. Using PacBio long reads, 98% of gaps were solved. In human chromosome 14 assemblies, FGAP reduced the number of gaps by 35%. All the inserted sequences were validated with a reference genome using QUAST. The source code and a web tool are available at http://www.bioinfo.ufpr.br/fgap/.

## Background

Low-cost and high-throughput sequencing technologies have increased exponentially the amount of sequence data available. The development of these technologies combined with advances in computer algorithms provided a large number of sequenced genomes. However, more than a third of these genome sequences available in public databases remain as drafts and many other projects are still incomplete [1] because of limitations of short read second-generation sequencing and assembly processes. Sequencing errors, regions of high complexity and repeated sequences are the most common issues. The single molecule third-generation sequencing technology [2] solved some of these limitations with longer reads, but brought in others such as high error rate and higher cost. Thus, there is still a dependence on second-generation sequencing platforms. The vast majority of genomes available today were sequenced using short-reads and their assemblies can still be improved.

Developments of the finishing process, which comprise error correction, scaffolding and gap closing, did not follow the speed of sequencing technologies. One strategy to reduce the number of gaps is to obtain data from different sequencing technologies, aiming to reduce errors, compensate bias and improve quality and completeness of the genome sequence [3]. Another approach is to obtain alternative assemblies using the same raw data, but with different assemblers and parameters [4]. These strategies usually generate many datasets, which can be combined to improve the genome. Some methods such as GapCloser (a module of SOAPdenovo2 [5]), GapFiller [6] (not to be confused with [7]), IMAGE [8], FinIS [9] and CloG [10] were designed to reduce the gaps in genome assemblies using different approaches.

We propose an open-source software called FGAP, that aims to improve genome sequences by merging alternative assemblies or incorporating alternative data, analyzing the gap region and indicating the best sequence to close the gap.

## Methods

FGAP searches for sequences that overlap contig ends in proposed scaffolds. It needs at least two Fasta files to run: the draft genome assembly and one or more contig datasets (alternative assemblies, long reads, contigs). The algorithm aligns contig ends from the draft assembly against datasets, selects the alignments with given parameters, and chooses the best sequence to eliminate the gap.

**Figure 1:**
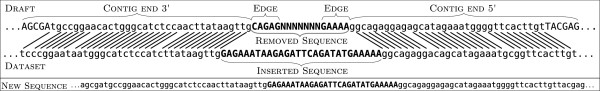
**Overview of a gap handled by FGAP.** Lower case characters represent the sequences aligned; diagonal lines represent the BLAST alignment.

FGAP uses nucleotide BLAST [11] algorithm to perform alignments. The program identifies unknown bases, represented as "N", and searches for identity in both sides of the gap against the datasets. The sequence at the edges of contigs may be ignored for these alignments since they are frequently of low quality (Figure 1). All contig ends are aligned separetedly. Alignments will be restricted by minimum score, maximum e-value and minimum identity. BLAST alignment parameters such as open gap, extend gap, match, mismatch, word size and the maximum number of alignments per query can be set. The maximum number of bases to be inserted or removed are also controlled. All these parameters will restrict the returned alignments, choosing the highest scored result. Sequences in the datasets are selected if they overlap the draft assembly’s contig ends and if they are within the defined length limits for the gap. Activating the negative gap option allows FGAP to solve gaps caused by overlapping contig ends.

Three assemblies of *Escherichia coli* str. K-12 substr. MG1655 were used to evaluate FGAP (Table 1). The data were obtained from the NCBI Short Read Archive (SRA) and consist of a paired-end Illumina HiSeq 2000 reads with insert size of 200 bp (SRR826451) and single-end 454 GS FLX reads (SRR057662), which were assembled by SOAPdenovo2 [5]. The draft genome sequence was assembled using both libraries. Two alternative assemblies were made, one for each set of reads, to be used in FGAP to close gaps of the *E. coli* draft assembly. One additional PacBio SMRT sequencing dataset (SRR811719) was used separately to evaluate the benefits of long reads.

**Table 1:** ***E. coli *** **assemblies**

	***k*-mer**	**Gaps**	**Sequences**	**Size (bp)**	**N50 (bp)**
Illumina(pe) + 454(se) [Draft]	81	123	41(s)/32(c)	4554392	172167
454(se) [Dataset]	99	0	12407(c)	6274970	531
Illumina(se) [Dataset]	81	0	564(c)	4615235	63640

Datasets were assembled with single-end reads, generating only contigs; pe: paired-end; se: single-end; s: scaffolds; c: contigs.

Two assemblies of the human chromosome 14 were also used (Table 2). The ALLPATHS-LG [12] assembly was used as a draft sequence and the CABOG [13] contig assembly was used as a dataset to close gaps. Both were assembled with three different sequencing libraries. All data for human chromosome 14 were obtained from the GAGE evaluation [4].

**Table 2:** Human chromosome assemblies

	**Gaps**	**Sequences**	**Size (bp)**	**N50 (bp)**
ALLPATHS-LG [Draft]	4307	418(s)	87688255	81646936
CABOG [Dataset]	0	3541(c)	86255201	46694

All data were obtained from GAGE evaluation [4]. s: scaffolds; c: contigs.

To validate closed gaps, we compared the sequence inserted from all closed gaps and their flanking regions against the reference genomes of *E. coli* K-12 [GenBank:NC_000913.2] with 4,641,652 bp and human chromosome 14 [GenBank:NC_000014.9] with 107,043,718 bp. Gaps are considered correctly closed when: 1) flanking regions align at least 40% of their length (based on the contig end length choosen for FGAP) with the reference, 2) the identity of the flanking regions and the inserted sequences are higher than a threshold (the same defined for FGAP), 3) the identity is greater than it was before gap closing (flanking regions without insertion). The NUCmer algorithm [14] was used to perform this validation.

We compared the results of FGAP with three standalone tools for gap closing: GapCloser [5], GapFiller [6], and IMAGE [8]. These programs rely on the identification of paired-end or mate-pair reads that map at contig ends and extend them by performing local assemblies to close gaps. All available libraries for each organism (1 for *E. coli* and 3 for human chromosome 14) were used as input to these tools. Two other approaches could not be tested: the FinIS [9] software relies on the graph generated by the assembler and does not support SOAPdenovo2 [5] assemblies, whereas the CloG [10] approach has not been implemented. Details of each program are in Additional file 1.

### Implementation

FGAP was developed in Matlab/Octave and can run indistinctly in both languages via source-code. It also runs in compiled code (depends on MCR) or through the World Wide Web (available at [15]) without requiring any license. It uses BLAST+ 2.2.28 or higher. The algorithm runs in multiple rounds, necessary to prevent overlapping between gaps close to each other. This prevents modifications in the query sequence of the neighbor gap. The output consists of one Fasta and one log file per round, and a final statistics file. The log file contains the alignment information for both sides of each gap. The Fasta file contains the new sequence with the gap sequence reported in the log file. Changes are incremental in the output Fasta files.

## Findings

### Results

The number of gaps of the *E. coli* str. K-12 substr. MG1655 in the ordered scaffolds of the draft genome sequence dropped from 123 to 26, thus reducing the unknown regions by 78%. Furthermore, 96% (94/97) of the newly inserted sequences were in agreement with the reference *E. coli* K-12 genome sequence. Using only PacBio as dataset with the same parameters, 121 out of 123 gaps were closed and all of them were validated with the reference. Assemblies of the human chromosome 14 derived from two different programs were used to evaluate the performance of FGAP in a more complex genome. FGAP reduced the number of gaps by 35% (1527 gaps closed out of 4307) in this scenario.

### Software comparison

The comparison between the four programs is shown in Tables 3 and 4. In *E. coli* assemblies, FGAP, GapCloser, GapFiller and IMAGE had similar performances in terms of number of closed gaps, with the former being better in terms of local misassemblies, N50 size and contig number. The reads from PacBio (FGAP+Long) used as datasets allow to generate the best results with more complete genes, without local misassemblies, closing the majority of the gaps. This result is likely due to the presence of sequences in PacBio data absent in the Illumina and 454 datasets. On the other hand, it generated more indels (Additional file 1). It is noteworthy that FGAP outperformed all other softwares in terms of running time, being about two times faster than GapCloser. IMAGE performed poorly under our conditions, taking over 2 hours to run.

**Table 3:** **Software comparison in *E. coli *** **assembly**

	**Original assembly**	**FGAP**	FGAP + Long*	**GapCloser**	**GapFiller**	**IMAGE**
Nº of gaps	123	26	2	22	25	19
Nº contigs (≥ 1000 bp)	116	80	73	82	85	87
Local misassemblies	2	9	2	12	12	21
Complete + partial genes	4325 + 44	4377 + 34	4388 + 27	4375 + 35	4367 + 35	4389 + 67
N50	66462	132608	172148	112396	132608	110934
Inserted bases (bp)	-	3133	6931	6140	3098	37217
Execution time	-	42 s	2 m 55 s	1 m 19 s	19 m 23 s	2 h 46 m 29 s

The evaluation was performed by QUAST script v2.3 [16] (all metrics are in Additional file 1). The gene number was calculated based on a reference list with 4497 genes. *FGAP + Long stands for PacBio’s long reads used directly as datasets.

**Table 4:** Software comparison in human chromosome 14 assembly

	**Original assembly**	**FGAP**	**GapCloser**	**GapFiller**	**IMAGE**
Nº of gaps	4307	2780	2799	3690	3840
Nº contigs (≥ 1000 bp)	4386	2880	2930	3796	3979
Local misassemblies	215	296	386	339	301
Complete + partial genes	1064 + 497	1141 + 423	1121 + 448	1093 + 468	1078 + 488
N50	38359	61874	58014	45825	42385
Inserted bases (bp)	-	244379	1165698	421831	373900
Execution time	-	3 h 11 m	1 h 10 m	8 h 09 m	50 h 45 m

The evaluation was performed by QUAST script v2.3 [16] (all metrics are in Additional file 1). The gene number was calculated based on a reference list with 1655 genes.

FGAP and GapCloser performed similiarly when the human chromosome 14 assemblies were used (Table 4). However, FGAP was better in terms of local misassemblies, N50 size and identified genes. In this evaluation, GapCloser achieved the lowest running time but had the highest number of local misassemblies. GapFiller and IMAGE had the lowest number of gaps closed. Again, IMAGE performed poorly under our conditions, taking more then 50 hours to run.

In both cases the number of inserted bases by each software varied, probably due to differences in extension of gaps closed by each program, and it was also influenced by errors introduced by the different methods. Particularly, the IMAGE tool increased the genome size substantially more than the others, and also had the highest error rate. All comparisons were made with the scaffolds broken down into contigs.

## Discussion

We developed a new software for gap filling that can be helpful for genome sequence finishing. FGAP automatically integrates various datasets into a draft genome, an approach that differs from the extension of contig ends based on paired read information. The flexibility of input data is beneficial, since it can use different sequencing technologies or different assemblies and does not rely on paired-end or mate-pair data. Programs such as GapCloser, which was projected to work with Illumina data only, or FinIS, which requires a specific assembler, have more restricted use.

Compared to available tools, FGAP is the only one with a self-explained, human readable and complete output that shows every sequence inserted in each gap, their relative position and alignment. This output can be useful for further analysis. Furthermore, it was the fastest program tested on small genome sequences and can run in a notebook. FGAP is the only tool tested that has support for long reads from third generation sequencing. It is also available on the web, which is an even easier way to access the program. Only FGAP, GapCloser and IMAGE are freely available.

## Conclusion

We show that FGAP is an efficient tool to find regions to fill gaps of draft genome sequences. The tool demands low computational resources, the results can be easily analyzed by the output generated, and it can be used for small or large genome assemblies. FGAP can effectively reduce the effort to improve draft genome sequences in few steps, minimizing the number of unknown regions for human evaluation and reducing the need to obtain new data. In addition, FGAP has been successfully used to close gaps of draft sequences of several bacterial and fungal genome projects.

## Availability and requirements

**Project name:** FGAP;

**Project home page: **http://sourceforge.net/p/fgap/;

**Operating system(s):** Platform independent;

**Programming language:** Matlab (R2012a) or Octave (3.6.2);

**Other requirements:** BLAST+ 2.2.28 or higher (blastn and makeblastdb) and MCR - Matlab Compiler Runtime v7.17 (only for compiled version);

**License:** The MIT License (MIT)

## Competing interests

The authors declare that they have no competing interests.

## Authors’ contributions

VCP developed the code, validated the results and wrote the manuscript; HF tested the tool and revised the manuscript; VW contribute to the prototype; MBR revised the manuscript; FOP revised the manuscript; EMS contributed to the concepts and revised the manuscript; RTT proposed the concept and designed the prototype. All authors read and approved the final manuscript.

## Supplementary Material

Additional file 1 Additional parameters used, detailed computational specifications, complete report from QUAST comparison and table of features comparing standalone softwares for gap closing. Click here for file
