# Supplementary material for: FGAP: an automated gap closing tool
Source: BMC Res Notes. 2014 Jun 18;7:371. doi: 10.1186/1756-0500-7-371 (PMC4091766; doi:10.1186/1756-0500-7-371)
Supplement: Additional file 1 — Additional parameters used, detailed computational specifications, complete report from QUAST comparison and table of features comparing standalone softwares for gap closing. [file 1756-0500-7-371-S1.pdf]

Additional File 1

Additional Parameters

The default values were used to other parameters that are not cited

E. coli str. K-12 substr. MG1655

FGAP (v1.7):
minScore: 25; maxEValue: 1e-07; minIdentity: 70; contigEndLength: 300; edgeTrimLength: 0; maxRemoveLength: 500; maxInsertLength: 500; positiveGap: 1; zeroGap: 0; negativeGap: 0; gapChar: N; blastAlignParam: 1,1,1,-3,15; blastMaxResults: 200;

GAPCLOSER (v1.12):
max. read length: 101;
(lib) avg_ins: 200;

GAPFILLER (v1.11):
(lib) insert size: 200; min. error: 0.25; orientation: FR;

IMAGE (v2.33):
iterations: 10; k-mer: 81; velvet insert length: 200;

Human chromosome 14

FGAP (v1.7):
minScore: 250; maxEValue: 1e-07; minIdentity: 70; contigEndLength: 3000; edgeTrimLength: 0; maxRemoveLength: 500; maxInsertLength: 500; positiveGap: 1; zeroGap: 0; negativeGap: 0; gapChar: N; blastAlignParam: 1,1,1,-3,15; blastMaxResults: 20; threads: 64;

GAPCLOSER (v1.12):
threads: 64;
(lib. frag) avg_ins: 180; rank: 1;
(lib. shortjump) avg_ins: 3000; reverse_seq: 1; rank: 2;
(lib. longjump) avg_ins: 35000; rank: 3;

GAPFILLER (v1.11):
threads: 64;
(lib. frag) insert size: 180; min. error: 0.25; orientation: FR;
(lib. shortjump) insert size: 3000; min. error: 0.25; orientation: RF;
(lib. longjump) insert size: 35000; min. error: 0.25; orientation: FR;

IMAGE (v2.33):
iterations: 10;
Obs: Only lib. frag was used; IMAGE does not have direct support to multi-thread but the smalt step was changed to run in 64 threads;

Computer specifications used to validation

64 x Intel(R) Xeon(R) CPU E7-8837 @ 2.67GHz with 512Gb RAM

QUAST report complete - *Escherichia coli* str. K-12 substr. MG1655

Assembly	ORIGINAL	FGAP	FGAP+Long	GAPCLOSER	GAPFILLER	IMAGE
# contigs (≥ 0 bp)	196	99	**75**	95	98	92
# contigs (≥ 1000 bp)	116	80	**73**	82	85	87
Total length (≥ 0 bp)	4551771	4554904	4558702	4557911	4554869	**4588988**

Total length (≥ 1000 bp)	4531809	4547032	4557786	4554321	4549678	**4585931**
# contigs	121	87	**73**	83	87	91
Largest contig	325897	414012	**414013**	414012	414012	347922
Total length	4535999	4552598	4557786	4555296	4551260	**4588868**
Reference length	4641652	4641652	4641652	4641652	4641652	4641652
GC (%)	50.74	50.73	50.74	50.74	50.73	50.75
Reference GC (%)	50.79	50.79	50.79	50.79	50.79	50.79
N50	66462	132608	**172148**	112396	132608	110934
NG50	63640	132608	**148525**	112396	132608	110934
N75	41331	57888	**61315**	57888	57972	55559
NG75	40919	56977	**59718**	57222	57888	54813
L50	19	12	**10**	12	12	13
LG50	20	12	**11**	12	12	13
L75	41	25	**22**	26	25	28
LG75	43	26	**23**	27	26	29
# misassemblies	**1**	**1**	**1**	**1**	**1**	3
# misassembled contigs	**1**	**1**	**1**	**1**	**1**	3
Misassembled contigs length	**107298**	185322	185318	185319	124502	128348
# local misassemblies	**2**	9	**2**	12	12	21
# unaligned contigs	0 + 0 part	0 + 0 part	0 + 0 part	0 + 0 part	0 + 0 part	0 + 0 part
Unaligned length	0	0	0	0	0	0
Genome fraction (%)	97.714	98.084	98.177	98.108	98.033	**98.729**
Duplication ratio	**1.000**	**1.000**	**1.000**	**1.000**	**1.000**	1.002
# N's per 100 kbp	0.00	0.00	0.00	0.00	0.00	0.00
# mismatches per 100 kbp	**0.18**	4.11	3.49	1.19	1.16	6.35
# indels per 100 kbp	**0.07**	0.90	13.14	0.15	0.53	1.42
# genes	4325 + 44 part	4377 + 34 part	4388 + 27 part	4375 + 35 part	4367 + 35 part	**4389 + 67 part**
# predicted genes (unique)	4299	4291	4306	4303	4294	**4361**
# predicted genes (≥ 0 bp)	4301	4294	4308	4306	4296	**4368**
# predicted genes (≥ 300 bp)	3782	3789	3792	3793	3785	**3822**
# predicted genes (≥ 1500 bp)	572	574	573	**576**	574	574
# predicted genes (≥ 3000 bp)	51	51	51	51	51	**52**
Largest alignment	325897	414012	**414013**	414012	414012	347922
NA50	63640	115083	**133062**	110227	132608	107159
NGA50	63640	114132	**133062**	106887	132608	107159
NA75	41331	57888	**61315**	57888	57972	54813
NGA75	40919	56977	**59718**	57222	57888	54167
LA50	20	12	**11**	12	12	13
LGA50	20	13	**11**	13	12	13
LA75	41	26	**23**	27	25	29

LGA75	43	27	**24**	28	26	30

QUAST report complete - Human chromosome 14

Assembly	ORIGINAL	FGAP	GAPCLOSER	GAPFILLER	IMAGE
# contigs (≥ 0 bp)	4725	**3198**	3217	4108	4258
# contigs (≥ 1000 bp)	4386	**2880**	2930	3796	3979
Total length (≥ 0 bp)	84461062	84705441	**85626760**	84882893	84834962
Total length (≥ 1000 bp)	84346905	84606614	**85552356**	84788039	84748169
# contigs	4472	**2950**	2972	3861	4041
Largest contig	240773	295919	291565	**430178**	240787
Total length	84416099	84662221	**85584826**	84840143	84796166
Reference length	107043718	107043718	107043718	107043718	107043718
GC (%)	40.77	40.79	40.76	40.79	40.78
Reference GC (%)	40.83	40.83	40.83	40.83	40.83
N50	38359	**61874**	58014	45825	42385
NG50	28043	**44201**	43698	33807	31291
N75	20271	**32725**	29984	23693	22307
NG75	5755	10057	**10662**	7308	6973
L50	646	**410**	444	541	592
LG50	990	**625**	658	822	900
L75	1397	**888**	952	1182	1281
LG75	2843	**1759**	1792	2370	2534
# misassemblies	112	**89**	198	145	151
# misassembled contigs	103	**80**	176	130	140
Misassembled contigs length	**1411366**	1900234	3996310	2563115	2404531
# local misassemblies	**215**	296	386	339	301
# unaligned contigs	**0 + 47 part**	1 + 24 part	0 + 67 part	1 + 45 part	1 + 71 part
Unaligned length	**38159**	39547	46177	39012	45197
Genome fraction (%)	78.671	78.917	**79.667**	79.043	78.980
Duplication ratio	**1.002**	**1.002**	1.004	1.003	1.003
# N's per 100 kbp	54.60	54.44	**0.00**	54.32	51.86
# mismatches per 100 kbp	**67.32**	70.03	128.62	72.69	77.67
# indels per 100 kbp	**21.63**	22.76	23.18	22.12	22.46
# genes	1064 + 497 part	**1141 + 423 part**	1121 + 448 part	1093 + 468 part	1078 + 488 part
Largest alignment	240773	295919	291557	**430178**	240787
NA50	38336	**61643**	57660	45751	42219
NGA50	27960	**43957**	43287	33274	31076

NA75	20101	**32245**	29534	23434	22102
NGA75	5680	9786	**10330**	7148	6773
LA50	647	**411**	448	543	594
LGA50	992	**628**	663	827	904
LA75	1402	**893**	961	1190	1288
LGA75	2864	**1779**	1819	2395	2560

Features of standalone softwares for gap closing

	FGAP (v1.7)	GapCloser (v1.12)	GapFiller (v1.11)	IMAGE (v2.33)	FinIS (v0.2)
**Input data**	- Scaffolds (fasta)<br>- Pre-generated datasets (fasta)	- Scaffolds (fasta)<br>- Paired-reads with known insert size (fastq)	- Scaffolds (fasta)<br>- Paired-reads with known insert size (fastq)	- Scaffolds (fasta)<br>- Paired-reads with known insert size (fastq)	- Asembly output folder (contigs and graph)<br>- File containing scaffold information
**Output**	- Detailed output<br>- Stats file<br>- Closed scaffold (fasta)	- Closed scaffold (fasta)	- Stats file<br>- Summary for intermediare files<br>- Closed scaffold (fasta)	- Reports per iteration<br>- Closed scaffold (fasta)	- Stats file<br>- Closed scaffold (fasta)
**Method**	- Searches for sequences overlapping the ends of contigs of proposed scaffolds. The algorithm aligns the contig ends against one or more dataset sequences (contigs, alternative assemblies, long reads), selects the alignments with given parameters, and chooses the best to eliminate the gap.	- Uses the paired information to identify reads that belong to gaps in the proposed scaffold. The algorithm uses these reads to performs local assemblies and incorporates generated data.	- Uses the paired information to identify reads that belong to gaps in the proposed scaffold. The algorithm performs extension of contig ends through k-mer overlapping of identified reads and tries to extend the region.	- Uses the paired information to identify reads that belong to gaps in the proposed scaffold. The algorith performs local assemblies and incorporate generated data, extending contig ends to overlap the gap.	- Uses an exact quadratic programming formulation to analyse the graph generated in the assembly process, trying to find better paths through the graph and eliminate gaps.
**Sequencing technologies support**	Independent	All sequencing technologies with paired-end or mate-pair libraries (designed to Illumina data)	All sequencing technologies with paired-end or mate-pair libraries	All sequencing technologies with paired-end or mate-pair libraries	All sequencing technologies (assembled with Velvet or SOAPdenovo) with paired-end or mate-pair libraries
**Platform**	Platform independent (Web tool, compiled or Matlab)	Linux (only 64-bit)	Linux	Linux	Platform independent (depends on paid MOSEK C++ API)
**Availability**	Open-source	Freely-available	Paid (Free academic license)	Open-source	Open-source
